# NMR resonance assignments of the major apple allergen Mal d 1

**DOI:** 10.1007/s12104-016-9685-8

**Published:** 2016-05-11

**Authors:** Linda Ahammer, Sarina Grutsch, Martin Tollinger

**Affiliations:** Institute of Organic Chemistry, Center for Molecular Biosciences Innsbruck (CMBI), University of Innsbruck, Innrain 80/82, 6020 Innsbruck, Austria

**Keywords:** NMR resonance assignment, Mal d 1, Apple, Allergen

## Abstract

The major apple allergen Mal d 1 is the predominant cause of apple (*Malus domestica*) allergies in large parts of Europe and Northern America. Allergic reactions against this 17.5 kDa protein are the consequence of initial sensitization to the structurally homologous major allergen from birch pollen, Bet v 1. Consumption of apples can subsequently provoke immunologic cross-reactivity of Bet v 1-specific antibodies with Mal d 1 and trigger severe oral allergic syndroms, affecting more than 70 % of all individuals that are sensitized to birch pollen. While the accumulated immunological data suggest that Mal d 1 has a three-dimensional fold that is similar to Bet v 1, experimental structural data for this protein are not available to date. In a first step towards structural characterization of Mal d 1, backbone and side chain ^1^H, ^13^C and ^15^N chemical shifts of the isoform Mal d 1.0101 were assigned. The NMR-chemical shift data show that this protein is composed of seven β-strands and three α-helices, which is in accordance with the reported secondary structure of the major birch pollen allergen, indicating that Mal d 1 and Bet v 1 indeed have similar three-dimensional folds. The next stage in the characterization of Mal d 1 will be to utilize these resonance assignments in solving the solution structure of this protein.

## Biological context

In Central and Northern Europe as well as in Northern America the majority of all people who suffer from birch pollen allergy develop intolerance to certain kinds of fruits and vegetables. This IgE-mediated allergy is the result of initial sensitization to the major birch pollen allergen, Bet v 1, and subsequent immunologic cross-reactivity of the Bet v 1-specific antibodies with structurally homologous proteins in food (Geroldinger-Simic et al. 2011). Among the most frequent triggers of birch-pollen related food allergies are apples. Indeed, clinical studies showed that more than 70 % of individuals that are sensitized to birch pollen develop allergic reactions when consuming apples (Ebner et al. 1991; Geroldinger-Simic et al. 2011). Such cross-reactivity of allergens typically triggers oral allergic sydromes (OAS) such as itching and swelling of lips, tongue and throat (Mari et al. 2005).

The allergic reaction against apples (*Malus domestica*) is attributed to the major apple allergen, Mal d 1 (Vanek-Krebitz et al. 1995). Mal d 1 has been found in both pulp and peel of apples at variable concentrations in different cultivars with higher concentrations present in the peel (Matthes and Schmitz-Eiberger 2009; Pagliarini et al. 2013). In addition, the amount of Mal d 1 in apples depends on storage conditions after harvest and storage time (Kiewning and Schmitz-Eiberger 2014). The biological role of Mal d 1 remains, however, unclear. Like its homologue from birch pollen Bet v 1, Mal d 1 belongs to the group 10 of pathogenesis-related (PR) proteins (Fernandes et al. 2013). PR-10 proteins have a molecular mass of ca. 18 kDa and fold into a seven-stranded, highly curved antiparallel β-sheet (β1–β7) along with two consecutive short α-helices (α1, α2) and a long C-terminal helix, α3. In plants, PR-10 proteins are activated in response to different kinds of abiotic and biotic stress and a possible role of these proteins in plant defence responses to pathogens has been discussed (Fernandes et al. 2013).

Mal d 1 was first isolated, characterized and cloned in 1995 (Vanek-Krebitz et al. 1995). A range of isoforms and variants have subsequently been identified that are encoded by a large gene family (Mal d 1.0101–Mal d 1.0403, www.allergen.org). Sequence identities between Mal d 1 isoforms and Bet v 1 range from 55 to 68 % (Ma et al. 2006) and circular dichroism studies have showed that Mal d 1 is a folded protein with a secondary structure content that is comparable to that of Bet v 1 (Ma et al. 2006; Oberhuber et al. 2008). IgE antibodies specific for Bet v 1 are known to cross-react with Mal d 1 (Bohle 2006; Ma et al. 2006; Vanek-Krebitz et al. 1995) and vice versa (Haka et al. 2015), indicating the presence of similar IgE epitopes on the surface of these proteins. In addition, it has been shown that Mal d 1 is fairly sensitive to thermal denaturation (Somkuti et al. 2011) and prone for proteolytic degradation (Jensen-Jarolim et al. 1999; Kitzmüller et al. 2015).

These data suggest that Mal d 1 and Bet v 1 have a similar three-dimensional structures. However, experimental structural data for Mal d 1 are not available to date and NMR resonance assignments have not been published. Here we report the solution NMR backbone and side chain assignment of the recombinantly expressed Mal d 1.0101. This particular isoform of Mal d 1 was first cloned from Granny Smith apples (EMBL Genbank Database accession No. X83672) (Schoening et al. 1996).

## Methods and experiments

### Protein expression and purification

The DNA of Mal d 1.0101 (GenBank nucleotide code X83672, protein code CAA58646) was cloned into the expression vector pET28b by using the enzymes NcoI and XhoI. The internal NcoI cleavage site was eliminated by silent amino acid mutation for His140. pET28b is a 5368-bp low-copy plasmid with kanamycin resistance. The target DNA is transcribed by T7 RNA polymerase that is under control of the *lac*UV5 promoter. The plasmid was transformed into E. *coli* BL21 Star (DE3) according to the heat shock protocol. 100 ml of LB medium suppl. containing 25 µg/ml kanamycin was inoculated with a single bacterial colony and incubated at 37 °C overnight. The starter culture was spun down at 1575 × g and the cell pellet of 50 ml starter culture was resuspended in 1 L freshly prepared M9 minimal medium. For ^15^N and ^15^N/^13^C-labeling the expression was carried out in M9 minimal medium with 25 µg/ml kanamycin containing ^15^NH_4_Cl (Cambridge Isotope Laboratories) and ^13^C_6_-d-glucose (Sigma-Aldrich). The culture was grown at 37 °C to an optical density between 0.6 and 0.8 and overexpression of Mal d 1 was induced by adding IPTG (isopropyl-beta-d-thiogalatopyranoside) to a final concentration of 0.5 mM. The cells were incubated overnight at 16 °C and afterwards harvested by centrifugation at 2050×*g* for 20 min. The cell pellets were resuspended in 25 mM imidazole, 0.1 % (v/v) Triton X100 and 0.5 M urea. After three cycles of freezing and thawing with liquid nitrogen and a 37 °C warm water bath, DNAse I was added to a concentration of 1 µg/ml. To lower the viscosity of the cell lysate, a final sonication step was performed. The lysate was spun down at 9500 × g for 30 min. The supernatant was collected and passed through a 45 µm filter before loading onto an anion exchange column (6 ml Resource Q column,GE Healthcare). The protein was eluted with a 60 ml gradient from 0 to 100 % buffer B (25 mM TrisHCl pH 7.5, 1 M NaCl) at a flow rate of 2 ml/min. The fractions containing Mal d 1 were concentrated to 2–3 ml using Amicon Ultra Centrifugal Filters with 3 kDa cutoff (Millipore). Finally, the protein was purified by size exclusion chromatography (HiLoad 16/600 Superdex 75 prep grade, GE Healthcare) with 10 mM sodium phosphate buffer pH 6.9. Purified Mal d 1 was analyzed by SDS-PAGE and electrospray ionization (ESI) mass spectrometry using a 7 Tesla Fourier transform ion cyclotron resonance mass spectrometer (FT-ICR MS) (Apex Ultra 70, Bruker Daltonics) with an attached ESI source. The amino acid sequence of Mal d 1 was confirmed by MS/MS. Of note, the MS data showed that in ca. 10–20 % of Mal d 1 the N-terminal methionine (Met1) is cleaved off.

### NMR spectroscopy

Protein concentrations for resonance assignment were 0.5 mM for ^15^N/^13^C labeled and 0.8 mM for ^15^N labeled samples in 91 % H_2_O/9 % D_2_O (v/v) at pH 6.9, 10 mM sodium phosphate, 7 mM or 11.2 mM L-ascorbic acid, respectively. NMR experiments were carried out at 25 °C on a 600 MHz Bruker Avance II + spectrometer equipped with a Prodigy CryoProbe and on 500 MHz Agilent DirectDrive spectrometer equipped with a room-temperature probe. For resonance assignment we used ^1^H-^15^N-HSQC, ^1^H-^13^C-HSQC, and three-dimensional HNCO, HNCACB, CBCA(CO)NH, (H)CC(CO)NH-TOCSY, H(CC)(CO)NH-TOCSY, C,C-edited and C,N-edited methyl NOESY and ^1^H^15^N-HSQC-TOCSY experiments. Data were processed using NMRPipe (Delaglio et al. 1995) and analyzed with CcpNmr (Vranken et al. 2005).

### Assignment and data deposition

**Fig. 1:**
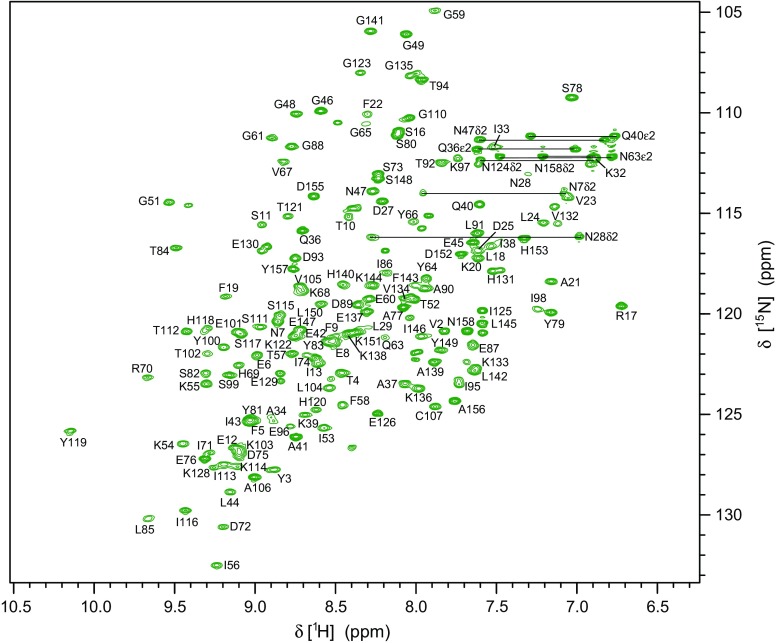
500 MHz ^1^H-^15^N-HSQC spectrum of Mal d 1.0101 (0.5 mM) at pH 6.9, 10 mM sodium phosphate, 7 mM L-ascorbic acid, 9 % D_2_O, 25 °C. *Horizontal lines* represent NH_2_ side chain resonances. Resonance assignments are available online at the BMRB repository (accession number 25968)

Backbone amide ^1^H-^15^N resonance assignment of Mal d 1.01010 was achieved for 144 (of 152 non-proline) residues corresponding to 94.7 % completeness (Fig. 1). C^α^ and C^β^ resonances were assigned for 98.1 and 97.2 % of all residues, respectively, while C’ assignments are 94.7 % complete. 96.5 % of H^α^ and 94.0 % of H^β^ resonances have been assigned. In addition, full assignments of side-chain amides in Asn and Glu (^15^N and ^1^H) and partial assignments of other side-chain resonances beyond β-positions (^1^H and ^13^C) have been obtained. All chemical shift data of Mal d 1.0101 have been deposited at the Biological Magnetic Resonance Data Bank (http:www.bmrb.wisc.edu) with BMRB accession number 25968.

**Fig. 2:**
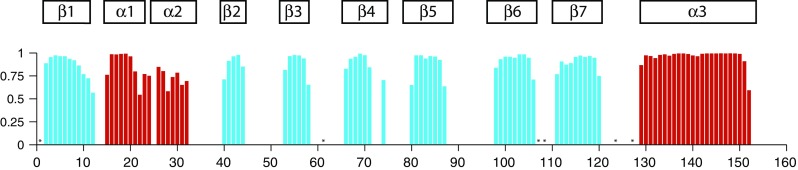
Secondary structure of the major apple allergen Mal d 1.0101 derived from H^N^, N, C^α^, C^β^ and C’ chemical shifts. The height of the *bars* reflects the probability of TALOS+ neural network secondary structure prediction. *Asterisks* indicate residues for which backbone amide NH resonance assignments are not available. For comparison, the secondary structure of Bet v 1 (α1–α3 and β1–β7) is indicated (Gajhede et al. 1996)

Using the Mal d 1.0101 H^N^, N, C^α^, C^β^ and C’ chemical shifts, a TALOS + prediction of the secondary structure elements of the protein was performed (Fig. 2). It is evident from the chemical shift data that Mal d 1.0101 contains seven β-strands (β1–β7) along with three (α1–α3) helices, in accordance with the canonical PR-10 fold (Fernandes et al. 2013). In total, Mal d 1 contains ca. 35 % beta and ca. 25 % helical structure, with striking similarities to the secondary structure of the birch pollen allergen, Bet v 1.0101 (Gajhede et al. 1996). Moreover, our chemical shift data agree well with secondary structure estimates from infrared (Somkuti et al. 2011) and circular dichroism (Ma et al. 2006; Roulias et al. 2014) studies of the isoform Mal d 1.0108 (99.4 % sequence identity with Mal d 1.0101).
